# Regulation of DNA replication by the S-phase DNA damage checkpoint

**DOI:** 10.1186/1747-1028-4-13

**Published:** 2009-07-03

**Authors:** Nicholas Willis, Nicholas Rhind

**Affiliations:** 1 Biochemistry and Molecular Pharmacology, University on Massachusetts Medical School, Worcester MA 01605, USA

## Abstract

Cells slow replication in response to DNA damage. This slowing was the first DNA damage checkpoint response discovered and its study led to the discovery of the central checkpoint kinase, Ataxia Telangiectasia Mutated (ATM). Nonetheless, the manner by which the S-phase DNA damage checkpoint slows replication is still unclear. The checkpoint could slow bulk replication by inhibiting replication origin firing or slowing replication fork progression, and both mechanisms appear to be used. However, assays in various systems using different DNA damaging agents have produced conflicting results as to the relative importance of the two mechanisms. Furthermore, although progress has been made in elucidating the mechanism of origin regulation in vertebrates, the mechanism by which forks are slowed remains unknown. We review both past and present efforts towards determining how cells slow replication in response to damage and try to resolve apparent conflicts and discrepancies within the field. We propose that inhibition of origin firing is a global checkpoint mechanism that reduces overall DNA synthesis whenever the checkpoint is activated, whereas slowing of fork progression reflects a local checkpoint mechanism that only affects replisomes as they encounter DNA damage and therefore only affects overall replication rates in cases of high lesion density.

## Introduction

DNA damage is a constant problem that cells must deal with to maintain viability. Proper duplication and segregation of undamaged genetic material to daughter cells is essential for survival. DNA damage may come from endogenous sources including reactive oxygen species produced by cellular metabolism, spontaneous depurination of DNA and replication fork collapse at various replication fork barriers, or from exogenous sources including ionizing and ultraviolet radiation. Failure to alter DNA metabolism to properly respond to damaged DNA can lead to genetic instability, resulting in cell death and, in multicellular organisms, oncogenesis [1].

To ensure each daughter cell receives a full complement of undamaged DNA, cells have evolved checkpoints. These checkpoints are surveillance mechanisms employed by the cell to detect and respond to DNA damage. They halt the cell cycle, allowing time to repair DNA damage before the crucial processes of DNA replication and chromosomal segregation [1,2]. Checkpoint deficiency leads to genomic instability as a result of failure to properly replicate, repair, or segregate damaged DNA.

Several checkpoints regulate the cell cycle. The G1/S and G2/M DNA damage checkpoints prevent cell-cycle progression into S-phase and M-phase, respectively. Additionally, the S-M checkpoint, also known as the replication checkpoint, prevents mitosis in the presence of arrested replication forks. The cell cycle targets of the G1/S, G2/M, and S-M checkpoints have been characterized [3]. In each case, they represent a global cellular response, in which checkpoint kinases regulate cell-cycle events that are distant from the initiating lesion. This model of checkpoint-as-global-regulator has been very useful for understanding checkpoint function, but may not completely fit the S-phase DNA damage checkpoint, also known as the intra-S checkpoint, which slows replication in the presence of DNA damage.

## The S-phase DNA damage checkpoint: global and local?

The S-phase DNA damage checkpoint is a bit different from the other DNA damage cell-cycle checkpoints. Instead of preventing a cell-cycle transition, this checkpoint reduces but does not absolutely halt DNA synthesis in the presence of damaged DNA during S-phase. In addition, there is not a strong correlation between checkpoint activity and DNA damage resistance. For these reasons, it has been suggested that the checkpoint may be more involved in accommodating and tolerating damage during replication than actually repairing the damage [4].

**Figure 1:**
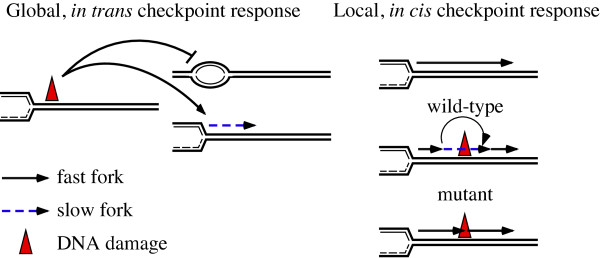
**Reduced Replication by Global and Local Mechanisms**. The checkpoint could act to slow replication using either global or local mechanisms. Slowing replication in response to DNA damage involves regulation of origin firing and replication fork progression. Origin firing is a global checkpoint response in which origins are prevented from firing that are not directly affected by DNA damage. In contrast, checkpoint regulation of replication fork progression may be a local or a global response to DNA damage. If global, all replication forks, both those encountering DNA lesions and those unperturbed by damage would be slowed. If local, only forks directly encountering damaged template would be slowed in a checkpoint-dependent manner.

The hallmark of the S-phase DNA damage checkpoint is the slowing of replication in response to DNA damage. Bulk replication can be slowed by inhibiting origin firing or reducing the rate of replication fork progression and both mechanisms appear to be used (Figure 1). Origin regulation is a global response in which factors act *in trans *to DNA lesions that may be far from the origins being regulated [5]. Checkpoint-dependent replication fork slowing may, like origin regulation, represent a global mechanism. If the checkpoint affects forks in a global manner, all fork progression would be slowed, both forks close to and far from sites of DNA damage. Alternatively, fork progression may be a local response to DNA damage. If checkpoint-regulation of forks acts *in cis *to DNA damage, only forks encountering DNA damage would be slowed. The contribution of fork slowing to total reduction in DNA synthesis would thus depend on lesion density. The idea that the checkpoint may act locally to slow replication at sites of DNA damage is consistent with checkpoint helping to coordinate replication and repair and to allow cells to tolerate damage during S-phase.

## Early studies on the effect of DNA damage on replication

Changes in the rate of DNA synthesis have been measured using a variety of bulk methods. Before the establishment of DNA as the critical target of radiation or even the genetic material, it was shown that replicating cells are more sensitive to ionizing radiation (IR) than stationary cells [6]. Furthermore, cells irradiated during the first third of the growth cycle (G1 and S-phase) are more sensitive to IR than cells irradiated thereafter [7]. Von Euler and Von Hevesy demonstrated that IR reduced incorporation of radiolabeled phosphate in treated cells [8]. This reduction was found to be DNA specific and IR exposure shown to reduce incorporation of deoxynucleotides into nascent DNA molecules [9-12]. With the discovery that eukaryotic genomes were duplicated by the combined activity of many replication origins and forks, investigators began to explore how cells slowed DNA synthesis in response to DNA damage, the central question being how the regulation of origin firing and fork progression contributes to reducing the rate of DNA synthesis in treated cells [13,14].

The first efforts to deconvolve origin and fork regulation used DNA fiber autoradiography and alkaline sucrose gradient centrifugation. Both techniques allow detection of new DNA synthesis by pulse-labeling replicating DNA. Fiber autoradiography permits direct visualization of DNA synthesis originating from multiple origins of replication on individual DNA fibers. Alkaline sucrose gradient centrifugation separates newly synthesized DNA of various sizes from the bulk of unreplicated DNA. The quantity and sizes of small species are used to measure the rates origin firing and fork progression. Experiments using these techniques suggested DNA synthesis was reduced due to inhibition of origin firing at low doses of UV or IR [15-18]. Concurrently, studies using high doses of radiation indicated both origin firing and fork progression were reduced in response to damage [19-23]. Supporting evidence for slowing of forks by UV damage was collected by electron microscopy showing asymmetric fork progression after UV irradiation [24].

**Figure 2:**
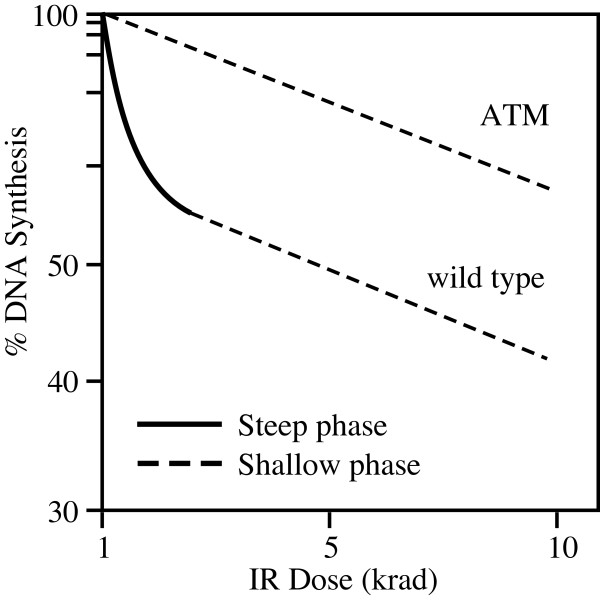
**Biphasic Dose Response to DNA Damage**. In response to DNA damage, cells reduce replication in a biphasic manner. In wild-type controls, initial reduction in DNA synthesis in response to low doses of ionizing radiation (IR) is steep (solid line) whereas declines in response to higher doses of IR is more shallow (dashed line). ATM checkpoint-kinase deficient cells do not display the initial steep reduction in synthesis in response to low doses of IR but do display similar response to wild-type controls at higher doses. Comparison between ATM and wild-type controls suggests that the shallow portion of the does-response curve may be checkpoint independent in nature. Figure adapted from [27].

Measurement of bulk DNA synthesis by radioactive label incorporation shows that cells respond in a biphasic manner to increasing doses of IR: a steep initial decline at low doses followed by a much more shallow reduction at higher doses ([25] and Figure 2). Estimates of fork progression and origin firing by alkaline sucrose gradient centrifugation indicated origin firing was more sensitive to DNA damage than fork progression [16,26]. Thereafter, it was broadly interpreted that the initial steep decline in replication to low doses of IR was due to prevention of origin firing while the shallow decrease at higher doses represent reduced replication fork progression (Figure 2).

## The checkpoint era

The study of checkpoints began with the realization that cell-cycle effects were not a passive consequence of DNA damage itself but an active regulatory response [2]. The first example of checkpoint regulation was in primary cells from patients suffering from Ataxia Telangiectasia (AT), a syndrome characterized by developmental defects, cancer predisposition and hypersensitivity to ionizing radiation. AT cells were shown to harbor a mutation in the Ataxia Telangiectasia Mutated (ATM) gene, and display a defect in the reduction of DNA synthesis in response to IR [25,27,28]. Elevated replication in the presence of DNA damage in AT cells was termed radioresistant DNA synthesis (RDS). RDS was subsequently found to be a common phenotype of checkpoint mutants from yeast to humans [29,30].

The AT RDS phenotype appeared to be primarily origin based. AT cells lacked the steep initial decline in DNA synthesis in response to low doses of IR. Analysis by both alkaline sucrose gradient centrifugation and autoradiography indicated that ATM mutants lacked control of origin firing in response to IR [16,28,31,32]. Thus, AT cells were proposed to display RDS due to an inability to prevent origin firing.

The role of ATM in regulating fork progression is less clear. First of all, fork slowing was observed only in response to high doses of radiation and most studies concentrated on the low dose results [28,33-35]. In a few cases, AT cells slowed replication fork progression as well as wild-type controls [33]. Indeed, both wild-type and ATM mutants display an identical shallow component suggesting the shallow portion of the dose response curve be a checkpoint-independent physical blockage of replication forks [27]. However, both genetic results and data obtained using physical techniques discussed later in this review suggest that fork progression is, in fact, regulated by ATM.

## Checkpoint regulation of origin firing

Checkpoint inhibition of origin firing is by necessity a global response to DNA damage. To prevent origins from firing, checkpoint components must act at origins distant from sites of DNA damage. Consistent with this idea, low doses of IR inhibit origin clusters not necessarily directly impacted by DNA damage and episomal DNA synthesis is prevented even when only nuclear DNA damaged by IR [16,18,36,37].

Significant progress has been made in elucidating the mechanism underlying checkpoint-regulation of origins in higher eukaryotes. Activation of either ATM or the ATM and Rad3-related (ATR) kinase prevents replication *in vitro *in *Xenopus *extracts [38,39]. In vertebrates, ATM activates Chk2 which then phosphorylates Cdc25A promoting its degradation [40,41]. Likewise, ATR activates Chk1, leading to the degradation of Cdc25A in response to UV [42,43]. Degradation of Cdc25A phosphatase prevents dephosphorylation and activation of the Cdk2-CyclinE complex thereby preventing the loading of the Cdc45 replication initiation protein onto origins and thus inhibiting origin firing [41].

Although checkpoint inhibition of origin firing is conserved between vertebrates and yeast [44,45], the mechanism is apparently not. In particular, Cdc25 is not required for replication slowing in fission yeast [46]. How origins are regulated in yeast by the checkpoint remains unknown.

Inhibition of origin firing is a general checkpoint response to replication fork stalling. Hydroxyurea (HU) treatment leads to nucleotide depletion, fork arrest, and replication checkpoint activation early in S-phase. Like the S-phase DNA damage checkpoint, the replication checkpoint prevents origin firing. Although the replication and S-phase DNA damage checkpoints are activated by different substrates, arrested replication forks and DNA damage, respectively, both checkpoints prevent origin firing via activation of the same checkpoint kinases. Gel-electrophoretic detection of replication intermediates at several origins showed that prevention of late origin firing upon HU arrest occurred in a checkpoint dependent manner [47,48]. Microarray analysis indicated that late-origin inhibition by the replication checkpoint is a genome wide phenomenon [49-53].

Checkpoint kinases regulate origin firing even in the absence of insult. AT cells were originally observed to replicate more quickly than wild-type [17]. Likewise, the rate of bulk DNA synthesis measured by radiolabel incorporation is increased in cells exposed to caffeine which inhibits both ATM and ATR prior to S-phase [54]. Furthermore, reducing Chk1 protein levels causes elevated origin firing during unperturbed S-phase [55,56]. Thus, checkpoints appear to limit origin firing under normal conditions.

## Checkpoint regulation of fork progression

In contrast to origin firing, fork slowing may represent a local checkpoint response to DNA damage. It is possible that checkpoint regulation of fork progression is a global mechanism in which, upon checkpoint activation, the rate of progression of every fork is slowed (Figure 1). However, we prefer a local model in which forks slow *in cis *to DNA damage for the following three reasons. First, there is no evidence that checkpoint activation can slow fork progression *in trans*. Second, there is no reason to believe that slowing in the absence of encountering damage would be beneficial to a replication fork. And third, as described below, there is evidence that the degree of slowing is correlated with the density of DNA damage. Therefore, we imagine a scenario in which forks transiently pause at sites of damage in a checkpoint-dependent manner. Each individual pause would not significantly delay replication, but the cumulative effect of forks pausing at many sites would lead to an overall reduction in fork rate. Therefore, even though the overall effect is referred to as 'fork slowing', we propose that forks are never slowed, per se; either they are replicating normally or they are transiently paused at sites of damage. This pausing would be checkpoint-dependent, since in checkpoint mutants replication is not slowed. It should be noted that although in our model fork slowing is a local, *in cis *effect, the checkpoint signaling involved need not be. For instance, forks encountering damage may activate checkpoint kinases that can act globally to inhibit replication origins or to regulate other forks, but such global signaling would slow only those forks which are also encountering DNA damage.

The checkpoint-dependent pausing could allow a number of responses to DNA damage: the replicative polymerases could be exchanged for translesion DNA polymerases allowing error-prone replication through the damage, the DNA lesion could be repaired after replication fork regression or the paused fork may undergo replication-coupled recombination allowing bypass of DNA damage through sister chromatid exchange. In the absence of the checkpoint, the fork would not pause at damage, possibly bypassing it by downstream repriming.

**Figure 3:**
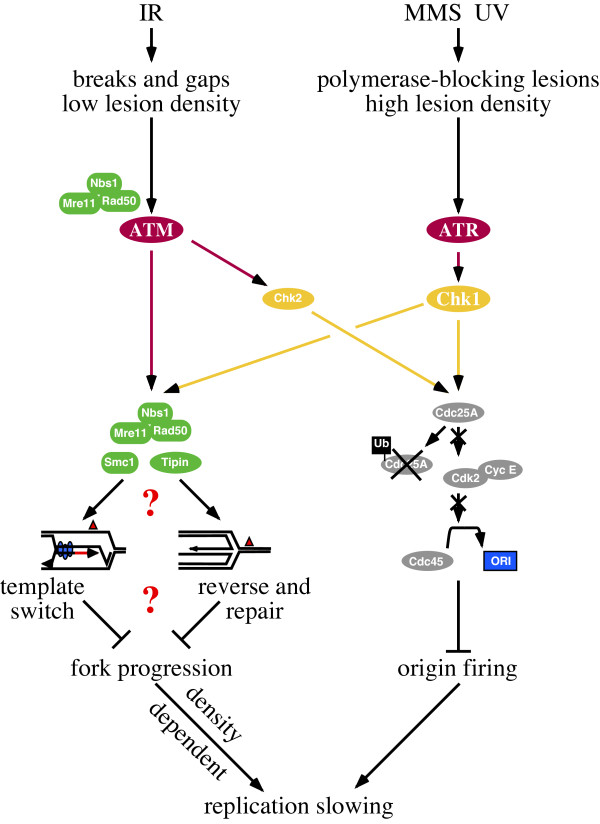
**A Model for Checkpoint-Dependent Slowing of Replication**. Different types of damage produce different densities of DNA lesions and therefore may have different consequences on replication. Both ATM and ATR regulate origin firing and fork response to DNA damage. However, the DNA damage determines the degree to which replication forks are slowed. IR produces a relatively low frequency of DSBs, which effect few forks while damaging agents producing a high density of bulky adducts including MMS and UV effect origins and many replication forks. Replication fork slowing may involve regulation of template switching or coordinating fork progression with repair.

Recent genetic evidence suggests that replication fork slowing contributes to checkpoint-dependent replication slowing. Two pathways downstream of ATM are required for robust slowing in response to IR (Figure 3). Mutations affecting either Chk2 or the Mre11-Rad50-Nbs1 (MRN) recombinational repair complex cause a partial RDS phenotype. Compromising both pathways induced robust ATM-like RDS [29,41,57]. Chk2 is required for prevention of origin firing but *Chk2 *mutants still display intermediate slowing. *MRN *mutants also display residual slowing even in response to IR fully capable of activating Chk2 and preventing origin firing [41]. These observations suggest the ATM RDS phenotype is due to defects in origin-dependent and independent, presumably fork slowing, events. Furthermore, increasing UV exposure causes an increase in the ATR-dependent bulk slowing of replication without further decrease in origin firing, suggesting a fork-dependent response [58].

Direct physical evidence for checkpoint-dependent slowing of fork progression has come from fluorescent DNA fiber analysis, an updated version of the fiber autoradiography. Using nucleotide analog labeling and fiber analysis, replicated regions on individual DNA molecules may be visualized. From this data, replication fork progression and origin firing frequency can be analyzed. This data explicitly shows DNA damage causes fork slowing, a possibility only indirectly supported previously by bulk assays. Both ATR and Chk1 are required to slow replication forks in response to UV and camptothecin (CPT) [59,60]. Besides the ATR and Chk1 kinases, vertebrate cells require additional components to slow forks in response to damage. The Timeless-Timeless Interacting Protein (Tim-Tipin) complex is a Chk1 target and Tipin is required for UV induced reduction of fork progression [59,61,62]. p53 is required for fork slowing in response to IR [63] and the Rad51 recombinase and its paralog Xrcc3 are required for fork slowing in response to CPT and UV [64]. These results are consistent with previous electron microscopy work showing asymmetric fork progression after UV irradiation [24].

Like regulation of origin firing, checkpoint kinases regulate fork progression even during unperturbed S-phase. Depletion of the ATR-Chk1 mediator Claspin or target Tim reduces fork progression in the absence of damage [55,59]. Presumably checkpoint kinases are able to act locally at spontaneously stalled forks without initiating a global checkpoint signaling response. In particular, Shimada et al., describe a threshold in checkpoint kinase activation which must be reached before a traditional, global checkpoint response is initiated [65].

In addition to checkpoint-dependent slowing, bulky DNA lesions can slow replication forks independently of checkpoint activity. Density shift experiments conducted in budding yeast followed replication of a long region initiated from a single origin. Results suggested replication fork progression was reduced in the presence of MMS in a checkpoint-independent manner [66]. Moreover, many studies that show checkpoint dependent slowing at moderate doses of damage also show checkpoint-independent slowing at higher doses.

## Stabilization of stalled forks, hints for fork slowing

The idea of the checkpoint as a fork modulator is consistent with the known vital functions of S-phase checkpoints in stabilizing stalled replication forks. First, fork stabilization allows for the completion of replication in the presence of damage. Two-dimensional gel electrophoresis and density shift analysis show checkpoint kinases prevent fork stalling and accumulation of unreplicated DNA in the presence of MMS [66-69]. Similar analysis has identified checkpoint-dependent fork stabilization of replication forks in response to UV [70]. Second, the checkpoint prevents excessive nuclease activity and ssDNA production at stalled forks. In budding yeast, electron microscopy shows checkpoint mutants display excess ssDNA and fork reversal upon HU arrest [71,72]. Unchecked Exo1 nuclease activity was found responsible for production of this ssDNA and checkpoint sensitivity to damaging agents which stall replication forks [73]. Lastly, checkpoint stabilization of stalled forks prevents fork breakage, a potentially lethal situation for the cell. In fission yeast, checkpoint mutants show increased incidence of asymmetric stalled fork breakage [74]. Complementing these studies, checkpoint mutants display increased incidence of recombination foci during HU arrest suggesting increased formation of DSBs [75-77]. A separation-of-function mutation in Mec1, the budding yeast ATR homolog, highlights the fact that fork stability is crucial to checkpoint-mediated cell survival. Like the null allele, Mec1-100 mutants display defects in origin firing, but they are able to maintain stable stalled replication forks and are extremely resistant to DNA damage [67,78]. Therefore checkpoint mutant sensitivity to S-phase insults appears to be due to fork destabilization and breakdown and not deregulated origin firing.

Fork slowing in the presence of damage appears to be a delicate balance between stalled fork stability and fork restart. Dynamic regulation of checkpoint kinase activity is required for efficient fork progression even when forks are slowed by DNA damage. Deactivation of Rad53, the budding yeast checkpoint effector kinase, is required for efficient replication fork progression in the presence of MMS [79]. Presumably the continued activity of Rad53 prevents replication fork restart and deactivation of Rad53 kinase is required for restart. This data suggests the checkpoint is able to maintain stalled forks in a replication competent state, preventing bypass of DNA damage, even after repair of the stalling lesion.

## Recombination, lesion bypass and fork slowing

Although genetic and physical evidence suggests checkpoint activation pauses replication forks, the exact mechanism by which the checkpoint accomplishes this feat is unknown. One possible mechanism for fork pausing involves recombination. Vertebrates require Rad51 for fork slowing in response to DNA damage [64]. Although the details appear to be different, recombination is also involved in slowing in yeast [70,80].

Although involvement of recombination in replication fork slowing differs amongst eukaryotes, recombination still appears to be important for replication fork metabolism. A possible mechanism involving recombination and fork pausing is polymerase template switching. Recombination intermediates or joint molecules are observed during replication [68,81-83]. Excessive production of these X-shaped intermediates has been postulated to indicate bypass of DNA damage during replication by uncontrolled template switching [84]. Checkpoint-dependent fork pausing may prevent recombination and template switching required for quick bypass of DNA damage. These X-shaped molecules produced by template switching accumulate in helicase and nuclease mutants during replication [81,85]. Thus proper fork metabolism involving control of polymerase template switching serves as a likely mechanism for checkpoint-dependent replication fork slowing [80].

Some data implicates that the restraint of recombination is important for slowing replication forks. Both budding and fission yeast helicase mutants *rqh1*Δ and *sgs1*Δ display hyper-recombinant phenotypes and S-phase slowing defects [80,82]. *sgs1*Δ mutants accumulate X-shaped intermediates during S-phase suggesting a correlation between failure to slow and unregulated template switching [81]. A number of proteins in fission yeast including the Rqh1 helicase, the MRN complex, the Rad2 flap endonuclease and the Mus81 endonuclease are required for slowing ([80] and Willis and Rhind unpublished data). All these proteins are involved in limiting or processing replication-dependent recombination events. Eliminating recombination by deletion of the central mitotic recombinase Rhp51 suppresses most of these mutants' slowing defects [80]. This epistatic relationship strongly suggests that preventing recombination promotes replication slowing.

The role of recombination in replication fork slowing is further supported by the conserved requirement of the MRN recombinational-repair complex for replication slowing in budding yeast, fission yeast and vertebrates [29,86-88]. In vertebrates, MRN is involved in both ATM and ATR dependent slowing pathways [89-91]. However, the role MRN plays in the checkpoint is complicated by its involvement in checkpoint signaling. MRN is required for ATM but not ATR activation, although primarily in response to low doses of IR [92]. We speculate that at low doses of IR, MRN activity would contribute to global checkpoint signaling through its role in ATM activation and therefore global ability of the cell to prevent origin firing. At high doses of IR or in response to UV, checkpoint activation is MRN-independent, but MRN contributes to slowing due to its local role on replication fork progression.

Like the regulation of origin firing, the phenomenon of replication fork slowing is conserved, although the mechanism seems to vary among eukaryotes. For example, components of the Replication Fork Protection Complex, namely Timeless/Swi1/Tof1, Tipin/Swi3/Csm3 and Claspin/Mrc1, serve different roles in stalled fork metabolism but are all required for some aspect of replication fork progression. In fission yeast, Swi1 is not required for slowing but is responsible for slowing defects displayed by other mutants. In contrast, both components of the vertebrate Tim-Tipin complex are required for slowing replication forks and fork progression in the absence of damage and, in *Xenopus*, the Tim homologue is required for fork restart [59,62,80,93,94]. Additionally, in vertebrates, Rad51 is required for slowing [64]. However, in fission yeast, recombination is not required for slowing in the presence of DNA damage, but is required for slowing defects displayed by several helicase and nuclease mutants [80].

## Lesion density may differentiate between origin-based and fork-based checkpoint responses

At first glance, yeast, frogs and mammals seem to differ in S-phase DNA damage checkpoint response to different damaging agents. However, differences in slowing may simply be related to the frequency of DNA lesions produced in these different systems. For example, vertebrates slow replication in response to low doses of IR while fission yeast do not [27,95]. In these model systems, exposure to IR prevents origin firing, demonstrating a global checkpoint response. These observations suggest that origin regulation is more important for slowing in vertebrates. We speculate that since replication takes far longer in vertebrate systems than in fission yeast (8 hours versus 20 minutes), the effect of global origin inhibition will have a greater impact on replication in vertebrates.

DNA lesion density may contribute to whether checkpoint-dependent fork slowing is manifest as slowing of bulk replication. Unlike global checkpoint inhibition of origin firing, which inhibits all origins once a threshold of checkpoint signaling is crossed, if fork slowing is local in nature, its contribution to bulk slowing would depend explicitly on the number of forks directly encountering DNA lesions. The greater the lesion density, the more often forks would pause and the greater the contribution of fork slowing to overall reduced DNA synthesis. Data from titration experiments using MMS shows replication slowing in fission yeast is directly related to the concentration of MMS used [80]. DNA fiber analysis also provides evidence that lesion density is important for replication fork response to damage. Merrick *et al*. observed in vertebrate cells that low doses of IR (1-5Gy), which cause 100s of lesions, prevented origin firing and did not slow forks but that MMS, which causes 10,000s of lesions, prevented origin firing and robustly slowed forks [96]. These observations suggest that DNA damaging agents reduce replication by different means; IR primarily prevents origin firing whereas MMS much more robustly slows replication forks. Furthermore, these observations support a direct correlation between DNA lesion density and the observed effect on replication fork progression. Differences between ATM and ATR regulated slowing may be due to the type of DNA damage these kinases respond to (Figure 3). ATM and ATR appear to promote replication slowing using the same mechanisms, a global prevention of origin firing and local slowing of replication fork progression. For example, the ATM-Chk2-Cdc25A and the ATR-Chk1-Cdc25A pathways limit origin firing in the presence of IR and UV induced damage, respectively. ATM and ATR also slow replication forks in response to IR and UV, respectively. However, UV causes many more fork-pausing lesions than IR, making ATR appear to have a more important role in fork slowing than ATM. Early confusion regarding the role of replication forks in slowing may be due to the local fork response being largely masked by the global origin response when replication was measured by bulk assay. At low doses of IR, ATM simply prevents origin firing without much measurable effect on replication fork progression [96]. Lack of replication fork slowing would not be due to the inability of ATM to regulate forks, but because IR does not produce enough lesions to effect many forks. The biphasic dose response curve described earlier may reflect this. All doses of IR effectively prevent origin firing in an ATM-dependent manner, but higher doses of IR may induce slightly more slowing because lesion density is moderately increased and more forks directly affected and slowed, also in an ATM-dependent manner.

## Conclusion

Despite some important differences between model systems, we propose checkpoint regulation of both origin firing and fork progression play important roles in checkpoint-dependent replication slowing. Direct evidence for both these mechanisms has recently been shown using fluorescent DNA fiber analysis. We propose that checkpoint activation prevents origin firing in a global manner but slows forks only when they encounter DNA damage. Thus, the contribution of fork slowing to the overall reduction in DNA synthesis is dependent on the density of DNA damage. The importance of proper checkpoint regulation of origin firing and fork progression in response to damage is emphasized by the fact that defects in the S-phase DNA damage checkpoint response are often associated with genomic instability, inability to tolerate DNA damage during replication and a predisposition to cancer development. Thus continued analysis of the mechanisms of the S-phase DNA damage checkpoint will undoubtedly produce interesting and important insights into how cells deal with DNA damage during S phase.

## Competing interests

The authors declare that they have no competing interests.

## Authors' contributions

NW and NR collaborated on the writing of this review.
